# Amyloid-like Structures in Marine Adhesive Proteins

**DOI:** 10.3390/md23090363

**Published:** 2025-09-19

**Authors:** Mariana Rodrigues Santos, Bárbara Joana Henriques, Romana Santos

**Affiliations:** 1 BioISI—Instituto de Biosistemas e Ciências integrativas, Faculdade de Ciências, Universidade de Lisboa, 1749-016 Lisboa, Portugal; mrosantos@ciencias.ulisboa.pt (M.R.S.); bjhenriques@ciencias.ulisboa.pt (B.J.H.); 2 Centro de Ciências do Mar e do Ambiente (MARE), ARNET—Aquatic Research Network, Departamento de Biologia Animal, Faculdade de Ciências, Universidade de Lisboa, 1749-016 Lisboa, Portugal; 3 Departamento de Química e Bioquímica, Faculdade de Ciências, Universidade de Lisboa, 1749-016 Lisboa, Portugal; 4 Departamento de Biologia, Faculdade de Ciências, Universidade de Lisboa, 1749-016 Lisboa, Portugal

**Keywords:** marine bioadhesion, permanent bioadhesives, non-permanent bioadhesives, functional amyloids, biomimetic adhesives

## Abstract

The formation of amyloid-like structures is commonly linked to neurodegenerative diseases, such as Parkinson’s and Alzheimer’s. However, proteins can form amyloid-like structures in non-pathological contexts, referred to as functional amyloids. We review the current knowledge on the evolutionary and ecological significance of the presence of proteins presenting amyloid-like structures in adhesive secretions (both permanent and non-permanent) produced by several marine organisms; in addition, we analyze the molecular and structural properties that make them well suited for this task and their possible biomimetic and industrial applications.

## 1. Introduction

Amyloid formation is frequently linked to diseases such as Alzheimer’s and Parkinson’s. However, some proteins can form amyloid-like structures, highly stable and insoluble protein assemblies characterized by a fibrillar morphology, a distinctive cross-β sheet structure and specific dye-binding properties, without leading to pathological plaque formation; these are often referred to as functional amyloids [1,2]. Unlike pathological amyloids, whose formation is an uncontrolled process where the oligomers/fibers formed can potentiate the fibrillation process (seeding effects) and the fibers can differ between themselves (polymorphism) [3], the formation of functional amyloids tends to be a more strict and controlled mechanism (no seeding or self-replication) with less structural variation [4,5].

Amyloid-like structures have been reported in different organisms, from bacteria [6], to algae [7], insects [8], humans [1,9] and numerous marine animals [10,11,12,13], where they serve specific functions, from being structural scaffolds to protection or substrate attachment.

Among their diverse functions, these amyloid-like structures play a crucial role in adhesion, an essential adaptation for marine organisms living in dynamic aquatic environments. In line with this adhesive function, a common feature of marine adhesive secretions is the formation of insoluble aggregates similar to amyloid-like structures. This characteristic provides stiffness and cohesion to the adhesive, enhancing its resistance to underwater conditions such as hydrodynamics, temperature, salinity, pH and microbial degradation [14,15]. In addition, amyloid-like structures in the adhesive material can serve as a precursor/catalyst for the polymerization and solidification of adhesive secretions, a phenomenon previously described for barnacle cement proteins [14].

The growing evidence of the presence of amyloid-like structures in marine adhesives incited us to review current knowledge on their evolutionary and ecological significance, their molecular and structural properties, their mechanisms of assembly, and their biomimetic and industrial applications.

## 2. Environmental Pressures Favoring the Development of Amyloid-like Structures in Marine Adhesives

Shoreline-dwelling marine organisms are subjected to forces exerted by currents and waves, such as drag (force in the direction of the flow), lift (force perpendicular to the direction of the flow) and acceleration (force that pulls the organism in the direction in which the water is accelerating). To survive in this environment and prevent dislodgment, many marine animals developed adhesion mechanisms that allow them to stay attached to the substrate, even under high hydrodynamism [16,17,18,19].

Generally, adhesion in marine organisms can be permanent or non-permanent [20,21]. Permanent adhesion is characteristic of sessile organisms, such as barnacles, functioning mainly as position maintenance. These animals stay attached to a substrate their entire adult lives, which means that they need a very strong adhesion mechanism to maintain their position despite the varying conditions of the surrounding environment. For organisms with rigid structures (i.e., shells), it is also important that the adhesive has a degree of elasticity, as it must resist the actions of tides and waves, while mitigating the propagation of cracks or other types of damage to the adhesive joint or the organism itself [22]. Permanent adhesive secretions are composed almost exclusively of proteins and harden to form a long-lasting cement [19,21].

Non-permanent adhesion, on the other hand, can be transitory, instantaneous or temporary [19,21]. The adhesive secretions from animals such as barnacle larvae, flatworms and echinoderms (sea cucumbers, sea stars and sea urchins) are usually a mix of proteins and glycans (generally in a ratio of 2:1) and have a higher degree of hydration when compared to permanent adhesives, which means that their adhesive secretions can also be in the form of hydrogels [20,21,23,24,25,26,27,28]. In this case, the adhesive’s main functions are attachment, locomotion and food collection [19,21].

Marine adhesives also need to be adaptable and to cope with different surface chemistries and environmental conditions (such as pH, salinity and current strengths) that can change over time but also according to the geographical location the animal lives in [19,29,30].

The above-mentioned factors likely favored the convergent evolution of proteins that present particular molecular characteristics and are prone to forming amyloid-like structures as recurring components of marine adhesives.

**Figure 1:**
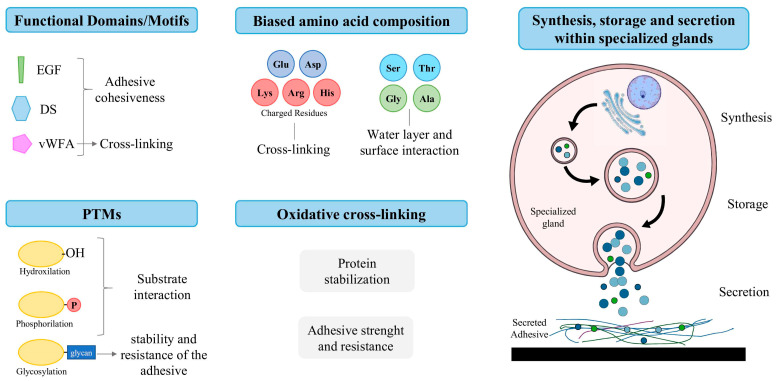
Schematic view of key molecular elements in marine adhesive proteins and their functions in adhesion process. Abbreviations: Ala, alanine; Arg, arginine; Asp, aspartic acid; DS, discoidin domain; EGF, epidermal growth factor domain; Glu, glutamic acid; Gly, glycine; His, histidine; Lys, lysine; Ser, serine; Thr, threonine; vWFA, von Willebrand factor type A domain. Images adapted from Servier Medical Art https://smart.servier.com/, licensed under CC BY 4.0 (https://creativecommons.org/licenses/by/4.0/, (accessed on 21 July 2025)).

In terms of key molecular elements (Figure 1), marine adhesives contain adhesive proteins that vary widely among species and adhesion types, yet share several conserved features:

1. Functional domains/motifs, such as epidermal growth factor (EGF) domains, which possess metal ion binding sites, and phosphotyrosine residues, which are associated with protein–metal and protein–protein interactions, as well as the cohesion of adhesive secretions [15,31,32,33]; von Willebrand Factor type A (vWFA) domains, thought to be responsible for cohesiveness and cross-linking [15,32,34,35]; and discoidin (DS)-like domains, linked to protein–protein and protein–carbohydrate interactions [36].
2. Post-translational modifications (PTMs), such as hydroxylation, phosphorylation and glycosylation [15]. Hydroxylated residues allow proteins to form hydrogen bonds with the substrate [15,37,38]; phosphorylated residues allow for the formation of ionic bonds with mineral and charged surfaces [15,39,40,41]; and glycosylation is thought to stabilize the conformation of adhesive proteins and confer resistance to proteolytic degradation [15,42,43].
3. Biased amino acid composition, such as the presence of charged residues (i.e., Lys, Arg, Glu, Asp and His), involved in protein cross-linking; and the abundance of Ser-, Thr-, Ala- and Gly-rich proteins, which are responsible for the interactions between substrate surfaces and the aqueous layer [15,19,23]. This is especially critical, as submerged surfaces are coated with a stable hydration layer that must be removed to allow for direct contact, given that this barrier tends to repel adhesives. Even after making contact, the presence of interfacial residual water can reduce its effectiveness by limiting the contact area between the adhesive and the substrate [44,45,46,47,48,49].
4. The occurrence of oxidative cross-linking, involving the formation of covalent disulfide bridges (–S–S–) between thiol (–SH) groups of cysteine residues, often mediated by enzymes like peroxidases [50,51,52]. The formation of these cross-links stabilizes the protein structure, enhancing adhesive strength, insolubility and resistance to degradation [25,39,53,54,55].
5. Biosynthesis in specialized cells within glands of the adhesive organs which have an optimized cellular environment (pH, temperature and presence of enzymatic cofactors), followed by packaging in secretory vesicles until exocytosis [15,25,43,56].

Likewise, some of the structural features of amyloid-like structures clearly benefit marine adhesive secretions:

1. Amyloid structures are known to possess high cohesiveness and mechanical strength (comparable to that of steel [23]), due to their modular nature [57] and the so-called “sacrificial bonds” [58]. These are weaker bonds between structural modules (i.e., β-sheets) of the amyloid-like structures that are preferentially broken when the fibril is subjected to any outside mechanical stress, preventing the backbone of the fibril from being exposed to stress, and potentially breaking and damaging the adhesive [57,58]. This structural characteristic is extremely important in the context of marine adhesion as it allows for the preservation of the integrity of the adhesive in dynamic environments.
2. Since amyloids self-assemble, it is thought that an amyloid-based adhesive can rapidly repair itself if damaged [58], which means that the sacrificial bonds mentioned above are likely replaced before any damage can come to the fibril backbone, allowing it to maintain the adhesion even when exposed to extreme circumstances, such as strong tides or predators.
3. Additionally, amyloids are known to be highly stable in water and degradation-resistant, which is a desirable trait for wet adhesives, where the constant presence of water could cause the deterioration of the adhesive due to increased permeability (plasticization), which could lead to their swelling, erosion and degradation [1,59,60].
4. The insolubility of amyloid-like fibrils, as they are composed of highly ordered packed β-sheets resistant to dissolution, can also be advantageous to marine adhesives. The adhesive will likely rapidly polymerize once secreted due to a difference in pH and/or ionic strength between the content of the secretory granules and seawater (pH 8), stimulating self-assembly of cross-β sheet structures that will not be dissolved and leading to a fast adhesion process [54,61], a characteristic often associated with marine adhesives.

## 3. Evidence of Amyloid-like Structures in Marine Adhesives

Amyloids are highly ordered insoluble structures that have a cross β-sheet motif [62,63]. The cross β-sheet motif is composed of a double β-sheet, where each sheet is made of β-strands stacked on top of each other in a direction perpendicular to the fibril axis through hydrogen bonding [62,63]. The interface between the two sheets is where the side chains of each of them interact; due to their complementary properties, this is a tight and dry region, referred to as the “steric zipper” [64]. The assembly of this type of structure depends on temperature, pH and solvent (water) [65]. Additionally, amyloid fibrils’ stability is ensured by a network of hydrogen bonds, hydrophobic interactions and π-π stacking interactions [63,66].

Currently, several methods are used to identify the presence of amyloids by exploiting their key structural features, such as elongated fibrillar morphology, low solubility and the characteristic cross-β-sheet motif [63]. The simplest approach is to use fluorophores like Thioflavin-T (ThT) or Congo Red that are frequently used to probe the presence of amyloid-like motifs or to follow amyloid fibril aggregation in vitro. It is, however, important to note that ThT recognizes the molecular groove commonly found on the surface of amyloid fibrils, but it can also bind to DNA, some polysaccharides or β-sheet-rich proteins. Therefore, fluorophore-positive staining of amyloid fibrils should be confirmed using additional techniques [67]. With respect to Congo Red, though its staining in bright field images suggests the presence of amyloid-like motifs, the appearance of apple-green birefringence under polarized light provides a stronger indication, as it is more specific to amyloid-like structures and more easily visualized, making it a better diagnostic tool [68].

Biophysical techniques such as circular dichroism (CD) and Fourier transform infrared (FTIR) can provide information on a protein’s overall secondary structure so they can also be used to prove the existence of amyloid structures. In CD, β-sheet-rich structures present a negative band centered at 220 nm, a characteristic of amyloid fibrils [69,70]. In the case of FTIR, it is more powerful as it can discriminate between different types of β-structures present in a protein; for example, fibrils and β-oligomers present a band around 1620–1625 cm^−1^ [69,70]. Both techniques are capable of following the conversion from native state to amyloid, though they do not give information on individual amyloid structures or morphology.

X-ray crystallography allows for the resolution of the cross-β sheet motif and provides information on the structure, periodicity and packing of amyloid fibrils [71], yet it requires sample crystallization, which is often not possible. Other advanced methods are available to obtain high-resolution structural information on amyloids without the need for crystallization, including solid-state nuclear magnetic resonance (ssNMR) and cryo-electron microscopy (cryo-EM), which allow for structure determination with atomic resolution [72,73,74,75,76]; atomic force microscopy (AFM), transmission electron microscopy (TEM) and scanning electron microscopy (SEM), which allow for high-resolution imaging of amyloid fibers at the nanoscale and the determination of physical properties, such as thickness and fiber diameter [77,78,79], and, in the case of AFM, provide additional information on their mechanical properties; and Raman spectroscopy or laser Raman spectroscopy [80,81], useful for analyzing the strain distribution within amyloid fibrils [82].

A combination of these techniques has been a major asset to identify amyloid-like structures in marine adhesives. To demonstrate their importance in marine adhesives, an extensive literature review was conducted to identify the various cases where this type of structure has been identified in marine-attaching organisms, as well as the techniques used to identify them and the type of sample that was analyzed (Table 1). For this review, an adhesive secretion or protein was considered putative amyloid-like if it met at least one (but often several) of the following criteria: positive ThT or Congo Red staining, periodic sawtooth force–extension curves observed by AFM, characteristic aggregation kinetics, a high prevalence of β-sheet content, fibrillar morphology, and/or protein sequence-based predictions consistent with amyloid-like properties (i.e., low complexity regions, intramolecular β-sheet structures) or a propensity for self-assembly.

The data indicate that amyloid-like structures are found in both permanent and non-permanent adhesives produced by various organisms (barnacles, flatworms, sea urchins and sea cucumbers). These structures are present in adhesive proteins secreted by distinct adhesive organs such as the adhesive glands of barnacles or Cuvierian tubules of sea cucumbers, and serve different functions, ranging from substrate attachment to defense.

### 3.1. Amyloid-like Structures in Permanent Adhesives

Mussels and barnacles stand out as the best studied permanent marine adhesives. While mussels depend on dihydroxyphenylalanine (DOPA)-containing proteins for adhesion, evidence of amyloid-like structures in the adhesive has only been reported for barnacles.

Barnacles are well-characterized sessile marine invertebrates and belong to the Crustacea subphylum (Cirripedia). Their main adhesive system is the secretion of a protein-based mixture that, once dried, forms a strong and permanent cement that is insoluble in water. This protein mixture is secreted from the cement glands through ducts that lead to pores in the baseplate of adult barnacles and allows them to adhere to almost all surfaces, making them a major target for antifouling technologies [14,30,94,95]. Once assembled and cured, the interior of the barnacle cement remains porous (pore size varies with substrate), which helps protect the joint from local damage and avoids the propagation of cracks in the cement, as any external stress applied to the joint is allowed to disperse in multiple directions [30]. Prior to adhesion, barnacles secrete a fluid containing lipids and reactive oxygen species (ROS) to strip the surface of organic matter and its water layer, creating a local hydrophobic environment conductive to adhesion [95,96].

Several barnacle cement proteins (CPs), usually referred to by their molecular weight (i.e., CP-100k), have been identified [30]. Barnacle cement proteins are generally characterized by an absence of PTMs, though glycosylation has been reported for CP-52k [30]. Interestingly, barnacles are the only sessile organism whose adhesive proteins do not possess DOPA residues.

Due to their unique sequences, barnacle cement proteins have few homologous counterparts in existing databases. However, in the last decade, homology has been reported with silk-associated proteins, such as fibroin from spider silk [85].

As a result, barnacle adhesive proteins have been mostly characterized by their amino acid composition and are classified into three groups: hydrophobic proteins (CP-52k, CP-100k and CP-114k), hydrophilic proteins (CP-19k-like and CP-43k-like proteins, including CP-68k) and CP-20k, a protein rich in charged amino acids (His, Asp and Glu) as well as cysteine [15,30,85,97].

Hydrophobic proteins are the most abundant (CP-100k and CP-52k account for 50% of all proteins in the cement) and, due to their insolubility in water, provide a stable framework for the adhesion of other cement proteins. They are involved in the self-assembly and curing of the cement [30,98].

Hydrophilic proteins (rich in Ser, Thr, Gly, Ala, Val and Lys), on the other hand, are located in the adhesive interface and facilitate interactions between the substrate surface and the water layer, acting like surface coupling proteins [30,97,99]. CP-19k, in particular, can adsorb onto multiple types of surfaces, known as a “multi-surface coupling” protein [99].

Lastly, CP-20k, another surface coupling protein, can form inter- and/or intramolecular disulfide bonds between adhesive proteins due to its cysteine residues, likely contributing to adhesive cohesiveness and insolubility [15,89,100]. It might also be responsible for adsorption onto calcareous surfaces [101]. Recent studies argue that CP-20k might be associated with the baseplate rather than with the cement [102].

**Figure 2:**
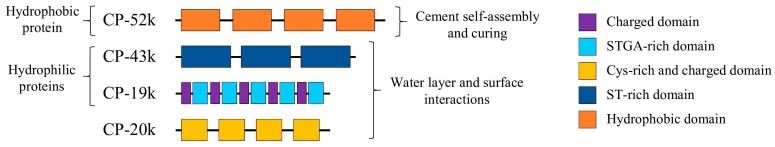
A schematic representation of the patterned sequence features of different barnacle cement proteins. Abbreviations: Cys, cysteine; STGA, serine–threonine–glycine–alanine; ST, serine–threonine. Modified from [103], under a Creative Commons CC BY license.

Barnacle adhesive proteins typically exhibit patterned sequence features (Figure 2), consisting of domains of residues with similar physicochemical properties, though not exact repetitions of each other [103]. This, coupled with the fact that some of these proteins have biases for specific amino acids, suggest that the key point may not be the primary sequence itself, but rather the presence of residues that facilitate the formation of particular bond types or specific secondary structures (i.e., amyloids) [100].

For example, CP-52k is composed of four hydrophobic domains [53], while CP-43k and CP-43k-like proteins are composed of three tandem ST (Ser-Thr)-rich domains [85]. A similar pattern is observed in CP-19k, composed of intercalating blocks of charged amino acids and STGA (Ser-Thr-Gly-Ala)-rich domains [41,54], or in CP-20k, made up of several domains that while not necessarily alike are enriched in charged and cysteine residues [104]. Interestingly, this pattern is not observed in CP-100k, which is mainly composed of hydrophobic residues without obvious sequence repeats, though its sequence seems to alternate between polar and non-polar residues [100,103].

This diversity in adhesion proteins is important for barnacle cement underwater adhesion, which relies on two mechanisms: (1) surface adhesion, which refers to the barnacle’s ability to strongly bind to different substrates and how the adhesive secretions interact with a given surface; and (2) bulk cohesion, which refers to the internal strength of the barnacle cement material, where the adhesive components interact with each other to confer stiffness and strength to the adhesive [103].

These mechanisms have been widely studied. Dickinson and colleagues [105] proposed that barnacle cement polymerization was similar to blood coagulation, based on the discovery of a transglutaminase in the non-cured form of barnacle cement (obtained from the cement glands) and the similarity of the barnacle cement nanostructure to the morphology of blood clots. Yet this hypothesis presents some flaws including the risk of contamination during sample manipulation and its inability to explain barnacles’ adhesion to a wide range of substrates [103,106].

Another proposal to describe these mechanisms is based on the identification of specific cement proteins. Kamino described that CP-20k and the hydrophilic proteins CP-19k and CP-68k facilitate non-covalent binding of the cement to the barnacle base shell and to the substrate, and that the hydrophobic proteins CP-52k and CP-100k are part of the bulk cement and polymerize via conformation-optimized intermolecular non-covalent interactions [97,99]. The weaknesses in this model include the insufficient evidence supporting the spatial distribution of cement proteins [103] and the potential misclassification of CP-20k as a cement protein, as previously mentioned [100,102].

More recently, So and colleagues [52] proposed an alternative model based on the discovery of enzymes such as peroxidases and lysil oxidases in barnacle cement. These enzymes promote covalent cross-linking of amyloid-like fibers, enhancing cohesion. According to this model, cement proteins first self-assemble into fibers through non-covalent interactions, which are then cross-linked in an enzyme-catalyzed process. The properties of these fibers may explain the high cohesiveness characteristic of barnacle cement [58,107]. Several barnacle cement proteins are thought to form amyloid-like fibrillar structures. Sequence analysis of CP-100k [87] and CP-19k [90,91] predicted the formation of amyloid-like β-sheets. Also, analysis of recombinant CP-52k [61] and CP-20k [89], both presenting amyloidogenic motifs, confirmed the formations of this type of structure (Table 1). Furthermore, these proteins contain residues with functional groups [i.e., hydroxyl (-OH), amino (-NH_2_) and charged groups] capable of interacting with the substrate, thereby promoting adhesive interactions, contributing to the high adhesive strength of barnacles’ cement [15,19].

**Figure 3:**
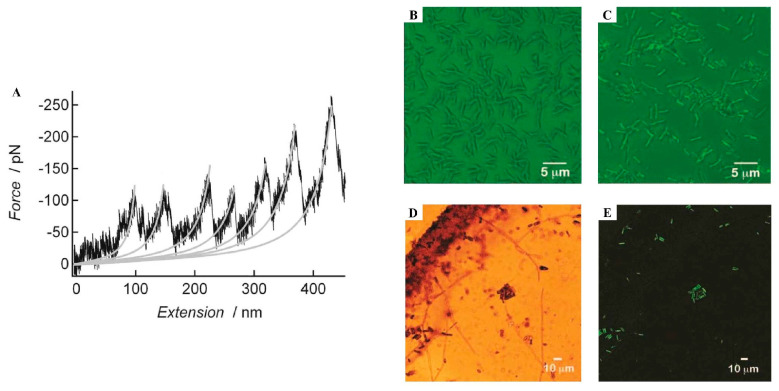
Morphological and structural characterization of adult barnacle cement from *Amphibalanus amphitrite*. (**A**) A single-molecule force spectroscopy sawtooth-like force–extension curve of the cement showing a profile characteristic of amyloid-like structures (black line). The worm-like chain behavior of a single protein chain without a modular nature is represented by the gray lines. (**B**) Confocal images of the adhesive in the absence of Thioflavin-T (control image) and positive staining of the rod-shaped structures in the adhesive by Thioflavin-T (**C**) and Congo Red staining in brightfield (**D**) and polarized light (**E**). Reproduced with permission from [84] Biofouling; published by Taylor & Francis, 2009.

Although this model is not yet widely accepted, since no cross-linked products have been identified in the barnacle cement [103], the increasing number of studies identifying amyloid-like structures in both adult barnacle cement (permanent adhesive) and larvae footprints (temporary adhesive) (Table 1) supports the model proposed by So and colleagues. These observations are consolidated by the fact that multiple methodologies were used, including the detection of cross β-sheets through Fourier transform infrared (FTIR) [10,83,84]; periodic sawtooth force–extension curves (Figure 3A) through AFM [84]; and staining with amyloid-specific dyes, such as ThT and Congo Red [10,84,86] (Figure 3B,C). Though there are macroscopic differences between species, they are all composed of nanofibers with amyloid-like characteristics [103].

Overall, the presence of amyloid-like motifs in several adhesive proteins in different species led to the conclusion that the formation of β-sheet structures is likely a key step in the curing/self-assembly process of the barnacle cement [91]. Unfortunately, due to the difficulties in extracting proteins from the cured cement, the information available for individual proteins and their role in adhesion is scarce. Nonetheless, there are several works, either using experimental methodologies or sequence-based predictions, that are important to mention in the context of this review.

In general, barnacle cement proteins have high isoelectric points (9.2 for CP-19k to 11.2 for CP-52k), which means that in seawater (pH 8) they are all positively charged proteins [91]. It has been theorized that the shift in ionic strength might trigger a change in protein folding, potentially leading to the formation of amyloid-like fibrils [61,90].

CP-52k is an unstable hydrophobic protein, predicted to consist mainly of loops and α-helices, with over half of its residues buried in the protein core (not solvent accessible) [91]. It has the highest number of aromatic residues, which contribute to its stiffness, insolubility [108] and ability to self-assemble through intermolecular interactions [61]. Several amyloidogenic segments in its sequence facilitate the formation of amyloid-like fibers in the marine environment [61].

CP-100k, also an unstable hydrophobic protein, is predicted to be composed of α-helices, β-sheet strands and loops [91] (predictions performed with Predict Protein [109]), though experimental studies on the species *Amphibalanus amphitrite* report conflicting results, ranging from predominantly β-sheets [87] to mostly random coils [84]. These discrepancies may stem from interspecific differences and analysis of incomplete sequences by Rocha et al. [91]. Like CP-52k, its residues are also mostly buried, but with fewer aromatic amino acids, making CP-100k more flexible (data obtained for *Pollicipes pollicipes* by Rocha et al. [91]), a trait that may help prevent crack propagation. *Megabalanus rosa* CP-100k also has a bias towards Ile, Val and Thr, which are amino acids associated with β-sheet formation [84,110].

CP-19k is a hydrophilic protein, composed of random coils and β-sheets, as shown by circular dichroism (CD) spectroscopy [111,112]. Structure predictions (performed with Predict Protein [109]) using sequences from several species indicate a higher loop content (48–74%) compared to β-sheets (26–43%), with most residues accessible to the solvent [91], which is consistent with its role as a surface coupling protein [101]. It also contains two conserved cysteine residues and a Gly-rich low-complexity domain (28 Gly in around 100 residues) [91], features commonly linked to cross β-sheet formation in amyloid structures [90,91,113].

Likewise, the hydrophilic CP-43k, generally considered stable, has most (91%) of its residues exposed to the solvent and its structure is predicted to be entirely composed of loops (data for *Amphilabalanus amphitrite*) [91].

Lastly, CP-20k, an unstable hydrophilic protein, rich in cysteine and charged residues (Asp, Glu and His) [104], is predicted to have most of its residues exposed to the solvent and arranged in loops (data from *Megabalanus rosa*, *Amphibalanus amphitrite* and *Fistulobalanus albicostatus*) [91]. However, NMR data on *M. rosa* revealed that this CP-20k presents three folded β-strand-rich regions connected by dynamic loops, enabling multiple conformations [89]. Furthermore, several basic residues exposed to the solvent near turns may mediate interactions with negatively charged surfaces [89], aligning with its proposed function as a surface coupling protein [55]. Additionally, 12 cysteine residues, located at the ends of β-strands, form disulfide bonds that stabilize both the amyloid-like β-sheets and a conserved β-motif (β7-β8), which acts as a seed for amyloid-like fibril formation [89].

### 3.2. Amyloid-like Structures in Non-Permanent Adhesives

Marine organisms that secrete non-permanent adhesives are not as well studied as those that use permanent adhesion. Nonetheless, amyloid-like structures have been detected in the adhesive secretions of barnacle larvae, flatworms and echinoderms (e.g., sea cucumbers and sea urchins).

As mentioned above, barnacles use both permanent and non-permanent adhesion depending on their life cycle stage. Cyprid barnacles use reversible adhesion to explore their surroundings and find a place to settle. Once settled, they attach through the secretion of a permanent adhesive (cyprid cement). Later, as sessile adults, this attachment is maintained through the secretion of barnacle cement [41].

During surface exploration, cyprids use two flat attachment discs covered in villi to “walk” along the surface through their successive attachment and detachment [114,115] and several antennular setae to sense the topological/biochemical/physicochemical characteristics of the surface. Cyprids tend to settle on surfaces they can strongly, but temporarily, adhere to [116], as well as surfaces where adult barnacles are already settled on, as proximity is needed for cross fertilization events.

The adhesion at this stage is achieved through the secretion of a bioadhesive by the unicellular antennal glands (in acorn barnacle cyprids) [114,115] or by the unicellular glands (in certain stalked barnacle cyprids) [117], which is then transported to the attachment site through ducts in the antennules. This adhesive is strong [109,110,111], fast (secured bond in seconds) [118] and reversible.

AFM and surface plasmon resonance (SPR) analysis of the footprints left behind by cyprid barnacles [92,119,120,121,122] confirmed the secretion of a protein-based adhesive and showed that the secreted adhesive is responsible for only one-third of the adhesion strength [119]. The remaining strength is speculated to be the result of physical surface contact mechanisms, due to the presence of tiny hair-like structures called microvilli on the attachment discs, which are thought to stick to surfaces using van der Waals forces (weak intermolecular attractions) in a similar manner as gecko spatula [119].

**Figure 4:**
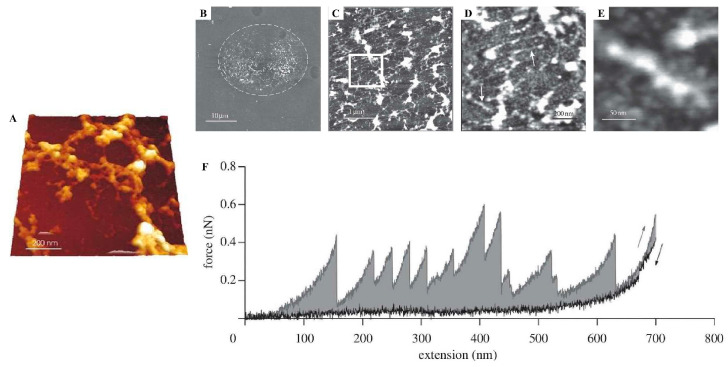
Morphological and structural characterization of cyprid barnacle adhesives from *Semibalanus balanoides* and *Amphibalanus amphitrite*. (**A**) A high-resolution 3D AFM micrograph of a protein adhesive of barnacle (*Semibalanus balanoides*) cyprid larva footprints in an aggregated fibrillar structure (scan size: 1 × 1 µm^2^ and z-range = 50 nm); (**B**) topographic AFM images of the adhesive of barnacle (*Balanus amphitrite*) cyprid larva footprints on NH_2_-terminated silanized glass in air (z-range = 100 nm); (**C**) different magnifications showing the extended conformation of nanofibrils across the surface and (**D**,**E**) allowing for visualization of single nanofibrils (highlighted in (**C**)); the arrows in (**D**) denote the spreading of a single chain across glass; (**F**) an AFM-based force spectroscopy sawtooth-like force–extension curve of the barnacle (*Amphibalanus amphitrite*) cyprid larva footprint showing multiple progressive unfolding peaks of footprint nanofibrils (gray arrow indicates extension curve, black arrow indicates relaxation curve). Reproduced with permission [93], The Journal of Adhesion, published by Taylor & Francis, 2009, and [92], Journal of the Royal Society Interface, published by The Royal Society Publishing, 2010.

Information regarding cyprid adhesive proteins is scarce, yet it is known that cyprid adhesives are mainly composed of hydrophobic and basic proteins, similar to the adhesive secretions of adult barnacles [121,123,124,125,126], but in contrast, they are often glycosylated [123]. Interestingly, two studies provide evidence for the presence of amyloid-like structures. Using AFM, it was demonstrated that cyprid barnacle footprints exhibit amyloid-like characteristics, such as an aggregated fibrillar structures (Figure 4A–E) and periodic sawtooth force–extension curves (Figure 4F), evidencing the existence of sacrificial bonds [92,93] (Table 1).

Among marine flatworms, the non-parasitic freshwater *Macrostomum lignano* are the best studied species in terms of their adhesive proteins. They produce a temporary adhesive to attach to the surface. This is achieved through a duo-glandular adhesive system composed of three different cell types (adhesive gland cell, releasing gland cell and anchor cell) that produce two proteins, the adhesive protein (ap1, responsible for surface adhesion) and the cohesive protein (ap2, responsible for the interface between the adhesive and the glycocalyx of the microvilli of the anchor cell) [127]. These proteins are usually conserved between species and present repetitive and Lys-Arg-rich regions, indicative of a propensity to form amyloid-like fibrils [128]. The adhesive also shares some similarities with that of barnacles and echinoderms, such as its high Gly and Ala content, the absence of DOPA residues, and low Tyr, Met and His content [129]. Till now, evidence for the presence of amyloid-like fibers has only been reported for the parasitic marine flatworm *Entobdella solae* [80] (Table 1), which also secretes a protein-based, highly insoluble, temporary adhesive [129]. This adhesive presents positive ThT stain, the detection of β-sheet structures by Raman spectroscopy, and observation of fibrillar morphology and periodic sawtooth force extension curves by AFM [80].

Concerning echinoderms, evidence of amyloid-like protein structures has been reported in sea urchin tube foot temporary adhesives [12] and in sea cucumber Cuverian tubules instantaneous adhesives [13] (Table 1).

Sea urchins use temporary adhesion for locomotion, feeding and attachment to the substrate. This strong adhesion is achieved by specialized adhesive organs (tube feet) followed by voluntary de-adhesion, carried out by a duo-glandular adhesive system composed of four different cell types (support, sensory, adhesive and de-adhesive cells) [21,130,131].

The secreted footprints contain both proteins and glycans, and the protein fraction is rich in certain amino acid residues (e.g., Gly and Asp) and presents adhesion-related functional domains (e.g., von Willebrand factor type D domain (vWFD), galactose-binding lectin domains, EGF-like domains or discoidin domains) [21,91,132,133]. In terms of post-translational modifications, both phosphorylation (e.g., in Nectin isoforms) [130] and glycosylation have been reported in several sea urchin adhesive proteins (e.g., Nectin, Alpha-tectorin, Alpha-macroglobulin, Myeloperoxidase). The main conjugated glycans identified were N-acetylglucosamine, its oligomers (mainly form of chitobiose units) and N-acetylgalactosamine [130,134,135,136,137]. Although the specific glycans and their abundance are variable between species, the presence of glycoproteins in sea urchin adhesives is conserved, indicating a possible role in the adhesive mechanism [136].

**Figure 5:**
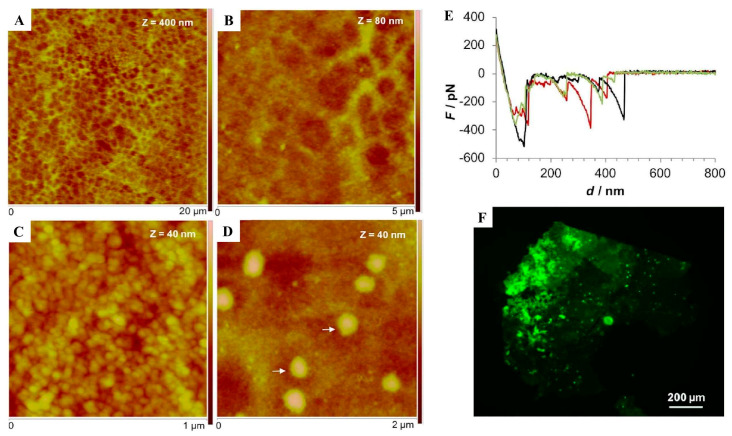
Morphological and structural characterization of the adhesive secretions of the sea urchin *Paracentrotus lividus*. (**A**,**B**) Peak force tapping (PFT)–AFM images of moist adhesive footprints of *Paracentrotus lividus* on mica, observed in air, showing the presence of an adhesive meshwork with a honeycomb appearance composed of (**C**,**D**) globular nanostructures (Z corresponds to image height); (**E**) sawtooth-like force–extension curves (Force in pN vs. distance in nm) of the adhesive footprints of *Paracentrotus lividus* on mica under native conditions (data obtained with a silicon nitride probe, *n* = 3; different colors correspond to the three independent experiments); (**F**) Thioflavin-T-stained adhesive footprints of the sea urchin *Paracentrotus lividus* on glass. Reproduced from Viana & Santos, Beilstein Journal of Nanotechnology, published by [12] Beilstein-Institut, 2018, under a Creative Commons CC BY license.

Adhesive footprints from the sea urchin *Paracentrotus lividus* were comprehensively studied by Viana & Santos [12]. As shown in Figure 5A–D, AFM images revealed that the footprints presented a meshwork-like structure with a honeycomb appearance, composed of globular nanostructures. Notably, the force–extension curves display a periodic sawtooth profile (Figure 5E), a hallmark of amyloid structures [12]. In addition, the footprints left on glass were positively stained by ThT (Figure 5F), a feature that had been observed for other marine adhesives (Table 1). Overall, this study suggests that sea urchin’s adhesive material contains amyloid-like structures, highlighting their importance in marine adhesion.

As for sea cucumbers, several species from the *Holothuriidae* family defend themselves against predators through ejection of the Cuvierian tubules which acquire instantaneous adhesive properties once expelled [138,139]. Cuvierian tubules are composed of an inner epithelium, a collagen-rich tissue layer and a mesothelium lining with adhesive granular cells [13].

The released adhesive is insoluble, acidic and mostly protein-based (rich in polar-charged and small side-chain amino acids) [140,141]. In the sea cucumber *Holothuria forskali*, the adhesive was reported to contain ~60% protein and ~40% carbohydrates, with the protein fraction being acidic and rich in small side-chain (e.g., Gly) and polar-charged amino acids—features linked to toughness and ionic/hydrogen bonding with surfaces. Uniquely, all adhesive proteins in sea cucumbers appear to share similar compositions, and phosphorylation of serine residues has also been observed [141,142].

The outer layer of the Cuvierian organ was positively stained with Congo Red, indicating the presence of amyloid-like fibrils, also observed through scanning electron microscopy. Several Cuvierian organ outer-layer adhesive proteins (COOLPs) were predicted to organize themselves in a cross-β motif, forming fibrils with amyloid-like properties (Table 1) [13]. These adhesive proteins (Hl-25083, Hl-25084, Hl-25088 and Hl-30757) have no orthologues in other marine species or functional domains but they are mostly composed of long tandem repeats and predicted to have a secondary structure composed almost exclusively of a series of intramolecular β-sheets [13].

## 4. Biomimetic Amyloid-like Marine Adhesive Proteins as an Inspiration for Adhesive Materials

Currently, clinical adhesives include synthetic glues (e.g., cyanoacrylates and polyethylene glycol), which can trigger immune responses, cure slowly and suffer from brittleness or swelling in wet environments [16,143,144,145,146]. Bioadhesives (e.g., fibrin and gelatin-resorcin aldehydes) are biocompatible but offer limited adhesive strength [147,148,149,150]. Importantly, both types perform poorly in wet conditions [103], making them less suitable for biomedical applications (e.g., surgical adhesives) or other cell-based biotechnological applications (e.g., cell culture, tissue engineering, 3D organ printing and lab-cultured meat production).

There is a growing interest in bioinspired adhesive alternatives that can rival synthetic glues in wet environments. Marine organisms exhibit exceptionally strong adhesion and resistance in aqueous salty media (similar to physiological fluids) and are usually more biocompatible than their synthetic counterparts, as they do not rely on organic solvents or toxic compounds [16]. Although limited, the information obtained from the few characterized marine adhesive proteins, along with data from adhesive secretions produced by marine organisms, is highly promising for the development of novel bioadhesive materials. Furthermore, as demonstrated throughout this review, these adhesive materials and proteins appear to exhibit amyloid-like features as a structural characteristic, which have already been shown to be important in the design of biomimetic materials. Additionally, amyloid-like proteins from other organisms have already inspired the design of several biomimetic materials, such as bioadhesives [151,152,153] and hydrogels [154,155,156]. A multi-functional wet-effective adhesive was obtained from fusing mussel foot proteins (Mfps) of *Mytilus galloprovincialis* with bacterial CsgA proteins, the major subunit of *Escherichia coli* amyloid curli fibers [153]. This bioadhesive combines the amyloid-like properties of the bacterial amyloid curli fibers (i.e., self-assembly into higher-order structures, strong adhesion and ability to display small sequences in fibril surface) with mussel Dopa residues (frequently present in some permanently attaching marine organisms) to form a new adhesive biomaterial that surpasses the adhesive strength of both its predecessors. Hydrogels have also been produced [154,155,156], taking advantage of the tunable mechanical properties and self-assembly ability of amyloid-like structures, to induce the formation of self-healing hydrogels with antibacterial properties. More examples of novel amyloid-inspired biomaterials can be found in [5,103,157,158].

Marine adhesive proteins are difficult to extract with high purity and in sufficient amounts for commercial use due to their low natural availability and their insoluble nature [103]. In order to circumvent this issue, researchers have shifted their focus to recombinant protein expression in an effort to maintain the key properties of marine adhesive proteins while being easier to mass-produce [159].

Currently, the most common method for obtaining large quantities of proteins, whether for biochemical characterization or for biotechnological applications, is heterologous production [160]. Among various host systems, the bacteria *Escherichia coli* is widely used due to its easy manipulation and low cost. However, protein expression in *E. coli* can sometimes result in low yields or protein misfolding, leading to the accumulation of insoluble inclusion bodies, which are more challenging to purify [161]. Notably, for certain technological applications, the formation of inclusion bodies can actually enhance production efficiency, as the target protein is more easily separated from cytoplasmic proteins [162]. Furthermore, to enhance recombinant protein production, fusion tags can be used to improve purification efficiency (affinity tags) or increase solubility and proper folding (solubility tags) [161]. Another approach is secreting proteins into the extracellular space using signal peptides or genetically modified bacteria, such as *Bacilus subtilis*, to simplify purification and improve yield [163].

For marine protein production, bacterial recombinant expression presents two major limitations. First is the lack of post-translational modifications, which are common in marine adhesive proteins [40,142,164,165,166,167]. This could be addressed using enzymes that modify specific residues post expression (e.g., hydroxylating Tyr to DOPA) [168], but this requires additional purification steps. Alternatively, for the case of DOPA, in vivo residue-specific incorporation during translation, as demonstrated by Deepankumar and colleagues [169], could be a strategy, avoiding extra purification. Second is the limited availability of complete genome data for most attaching marine invertebrates, making it difficult to identify and obtain full-length protein sequences for expression.

To overcome challenges in the recombinant production of marine proteins, researchers have explored the use of peptides inspired by marine adhesive proteins [14,61,88,170,171]. Due to their smaller size, lower molecular weight and lower structural complexity, peptides can be chemically synthetized, simplifying their production [103], and in some cases, the produced peptides retained the ability to self-assemble into amyloid-like fibrils [14,61,88,171].

Significant progress has been made in the recombinant expression of marine adhesive proteins, mostly on sessile organisms (e.g., barnacles) driven by their relevance to biofouling research and biotechnological applications. The first barnacle adhesive protein produced recombinantly was rCP-19k from *Megabalanus rosa* [101]. It was expressed in *E. coli* to elucidate its role in barnacle’s adhesive mechanism. The authors tested rCP-19k adhesive properties on several surfaces but no studies on its amyloid-like properties were carried out. Later, Liang and colleagues [111] produced a modified rCP-19k (containing two additional amino acid residues) which showed adsorption ability and high adhesive strength, and presented amyloid-like features, such as aggregation propensity and pH-dependent secondary structure, though the authors were unable to confirm this through ThT fluorescence [111]. Interestingly, another modified rCP-19k produced by Liu and coworkers was able to self-assemble into amyloid-like fibrils under various conditions, as confirmed by ThT fluorescence, TEM and FTIR, but its adhesive properties were not evaluated [172].

The remaining CPs are hard to recombinantly produce due to their high cysteine content and hydrophobicity [103]. Nonetheless, there are records of recombinant production of CP-20k from *Megabalanus rosa* [55,89,173], CP-43k from *Amphibalanus amphitrite* [85] and CP-52k from *Megabalanus rosa* [174]. Additionally, some studies have successfully enhanced the production yield of barnacle CPs through co-expression with other proteins, such as those naturally found in the extracellular environment, to create chimeric constructs [175,176]. Unfortunately, there is no information regarding their possible amyloid-like behavior.

In echinoderms, only one sea star-inspired recombinant protein has been produced. Lefevre and colleagues [177,178] recombinantly produced, in *E. coli*, several fragments of the *Asterias rubens* Sfp1 (sea star footprint 1) adhesive protein. A morphological analysis of the recombinant proteins and the effect of various ions (naturally present in the marine environment) on its adsorption properties was performed, but no evaluation of their amyloid-like properties was reported [177,178]. The authors found that in the presence of certain ions present in the marine environment (i.e., Na^+^, Ca^2+^ and Mg^2+^), the fragments tended to adhere to glass and/or polystyrene surfaces, and that the coatings formed were as non-cytotoxic to HeLa cells but also increased their proliferation, showing promise for future biomedical/biotechnological solutions.

In summary, amyloid-like structures have features that benefit marine adhesion (e.g., insolubility, cohesiveness, mechanical strength, self-healing and resistance to degradation), and they have been detected in the adhesive secretions of several marine organisms (with both permanent and non-permanent adhesion) through various methods. Amyloid-like proteins from different organisms have already inspired the design of new biomaterials for various applications, attesting to their usefulness in this area. However, there is still a lot to discover in the realm of marine amyloid-like adhesive proteins, especially because, as mentioned above, there is a large gap in identifying and sequencing proteins involved in marine bioadhesion.

The use of omics approaches such as genomics, transcriptomics and proteomics could help bridge this gap, as they provide complementary information, and due to advances in sequencing and mass spectrometry, these methodologies are faster, more accurate and more accessible. More research is needed to identify adhesive protein genes in different marine organisms, to elucidate how these genes are differently expressed under various adhesion conditions and to characterize the proteins secreted into the adhesive material, enabling us to accelerate the discovery of more wet-effective adhesive proteins that inspire the design of new biocompatible and biodegradable bioadhesives. Additionally, it is necessary to obtain high-resolution structures of these newly discovered adhesive proteins and the amyloid-like structures they form to understand the mechanisms of their formation and how they interact with different surfaces. So, in the coming years, deeper investigation and investment are essential to translate marine amyloid-based biomimetic adhesives to practical biomedical and biotechnological applications.

## Figures and Tables

**Table 1:** Evidence for the presence of amyloid-like structures in marine adhesives. For each organism, a brief description of the structural characteristic related to amyloid-like features is provided, along with the techniques used to observe these patterns. Abbreviations: AFM, atomic force microscopy; ATR-FTIR, attenuated total reflectance–Fourier transform infrared; CD, circular dichroism; CO, Cuvierian organ; COOLP, Cuvierian organ outer-layer protein; CP, barnacle cement protein; DLS, dynamic light scattering; MD, molecular dynamics; SEM + EDX, scanning electron microscopy–energy dispersive X-ray spectroscopy; ThT, Thioflavin-T.

	Organism	Methodological Approach	Amyloid-like Characteristics	Reference
**Permanent Adhesives**	Barnacle *Amphibalanus* *amphitrite* 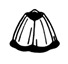	in situ ATR-FTIR	Cement containing mostly β-sheet (~50%) content, with minor α-helix, turned and unordered components; Detection of cross β-sheets and amyloid content in the cement.	[83]
		AFM SEM + EDX ThT stain Congo Red stain FTIR	Elastic cement with rod- and globular-shaped morphologies (nanoscale) of organic nature (AFM, SEM + EDX); the rod-shaped structures were positively stained by Thioflavin-T (ThT) and Congo Red, indicating 5% amyloid content. The cement contained both β-sheet and random coil content (FTIR) and showed a periodic sawtooth force–extension curve (AFM), characteristic of amyloid structures.	[84]
		Far-UV CD FTIR ThT stain AFM	Cement with both β-sheet (~30%) and disordered regions (~40%) (CD); composed of amyloid-like and globular structures (FTIR), thought to have around 28% amyloid content. The cement was positively stained by ThT and there was detection of nanofibrillar structures (AFM).	[10]
		SEM	Cement composed of a network of dense nanofibrillar structures.	[85]
		ThT stain	Cement positively stained by ThT.	[14]
	Barnacle *Lepas* *anatifera* 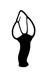	ThT stain	Adhesive glands and cement were positively stained by ThT.	[86]
	Barnacle *Megabalanus rosa* 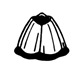	Bioinformatic tools	CP-100k protein was predicted to form amyloid-like β-sheets.	[87]
		DLS AFM SEM CD	Recombinant CP-20k peptides self-assembled in a pH and salt-dependent manner (DLS). There was formation of fibers made of bundles of nanofilaments (AFM, SEM). Changes in pH led to irreversible changes in secondary structure, with a possible increase in β-sheet content (CD).	[88]
		ThT stain CD AFM	Identification of amyloidogenic motifs (ThT) in recombinant CP-52k peptides that formed fibrillar entanglements and amyloid-like fibrils. Peptide conformation changed in response to pH and ionic strength increase (CD, ThT, AFM).	[61]
		NMR CD Bioinformatic tools	Recombinant CP-20k presented β-sheet and α-helix structures (CD, NMR). A highly stable and conserved motif—possible seed for fibrillization—was identified through MD simulations.	[89]
	Barnacle *Pollicipes* *pollicipes* 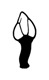	Bioinformatic tools	CP-19k was predicted to self-assemble into amyloid plaques under the appropriate environmental triggers.	[90]
		Bioinformatic tools	CP-19k has a long low-complexity Gly-rich region, which can be associated with β-sheet formation in amyloid development.	[91]
**Non-Permanent Adhesives**	Barnacle cyprids *Amphibalanus* *amphitrite* 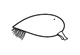	AFM	A footprint, porous in nature, with bundles of fibrils and individual nanofibrils. It showed sawtooth force–extension curves, characteristic of amyloid structures.	[92]
	Barnacle cyprids *Semibalanus* *balanoides* 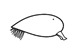	AFM	A footprint with an aggregated fibrillar structure. It showed a sawtooth force–extension curve, characteristic of amyloid structures.	[93]
	Flatworm *Entobdella solea* 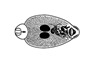	Raman spectroscopy AFM ThT stain	A footprint containing intermolecular β-sheets and strong intermolecular H-bonds (Raman spectroscopy) showed periodic sawtooth force–extension curves (AFM), characteristic of amyloid structures, and was positively stained by ThT.	[80]
	Sea urchin *Paracentrotus* *lividus* 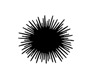	AFM ThT stain	A footprint presented a honeycomb-like meshwork of interconnected threads of globular nanostructures and periodic sawtooth force–extension curves (AFM), characteristic of amyloid structures. The footprint was positively stained by ThT.	[12]
	Sea cucumber *Holothuria* *leucospilota* 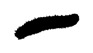	SEM Congo Red stain Bioinformatic tools	Detection of amyloid-like fibrils on the surface of the Cuvierian tubules (CO, adhesive organs) (Congo Red stain). Several COOLPs (Hl-25083, Hl-25084, Hl-25088, Hl-30757) were predicted to have a secondary structure composed of full intramolecular β-sheets.	[13]

## Data Availability

No new data were generated in this review.

## Abbreviations

The following abbreviations are used in this manuscript:

AFM	Atomic force microscopy
ATR-FTIR	Attenuated total reflectance–Fourier transform infrared
CD	Circular dichroism
CO	Cuvierian organ
COOLPs	Cuvierian organ outer-layer proteins
CP	Barnacle cement protein
Cryo-EM	Cryo-electron microscopy
DLS	Dynamic light scattering
DOPA	Dihydroxyphenylalanine
DS	Discoidin domain
EGF	Epidermal growth factor domain
FTIR	Fourier transform infrared
MD	Molecular dynamics
PFT-AFM	Peak force tapping–atomic force microscopy
PTMs	Post-translational modifications
ROS	Reactive oxygen species
SEM	Scanning electron microscopy
SEM + EDX	Scanning electron microscopy–energy dispersive X-ray spectroscopy
ssNMR	Solid-state nuclear magnetic resonance
TEM	Transmission electron microscopy
ThT	Thioflavin-T
vWFA	von Willebrand factor type A domain
vWFD	von Willebrand factor type D domain
